# Model-Based Simulation and Prediction of an Antiviral Strategy against Influenza A Infection

**DOI:** 10.1371/journal.pone.0068235

**Published:** 2013-07-09

**Authors:** Kye-Yeon Hur, Joon-Young Moon, Seung-Hwan Kim, Joo-Yeon Yoo

**Affiliations:** 1 School of Interdisciplinary Bioscience and Bioengineering, Pohang University of Science and Technology (POSTECH), Pohang, Republic of Korea; 2 Department of Physics, Pohang University of Science and Technology (POSTECH), Pohang, Republic of Korea; 3 Department of Life Science, Pohang University of Science and Technology (POSTECH), Pohang, Republic of Korea; 4 Department of Anesthesiology, University of Michigan Medical School, Ann Arbor, Michigan, United States of America; Melbourne School of Population Health, Australia

## Abstract

There is a strong need to develop novel strategies in using antiviral agents to efficiently treat influenza infections. Thus, we constructed a rule-based mathematical model that reflects the complicated interactions of the host immunity and viral life cycle and analyzed the key controlling steps of influenza infections. The main characteristics of the pandemic and seasonal influenza strains were estimated using parameter values derived from cells infected with Influenza A/California/04/2009 and Influenza A/NewCaledonia/20/99, respectively. The quantitative dynamics of the infected host cells revealed a more aggressive progression of the pandemic strain than the seasonal strain. The perturbation of each parameter in the model was then tested for its effects on viral production. In both the seasonal and pandemic strains, the inhibition of the viral release (*k_C_*), the reinforcement of viral attachment (*k_V_*), and an increased transition rate of infected cells into activated cells (*k_I_*) exhibited significant suppression effects on the viral production; however, these inhibitory effects were only observed when the numerical perturbations were performed at the early stages of the infection. In contrast, combinatorial perturbations of both the inhibition of viral release and either the reinforcement of the activation of infected cells or the viral attachment exhibited a significant reduction in the viral production even at a later stage of infection. These results suggest that, in addition to blocking the viral release, a combination therapy that also enhances either the viral attachment or the transition of the infected cells might provide an alternative for effectively controlling progressed influenza infection.

## Introduction

Influenza is the causative agent of the annual epidemics of seasonal flu, which has a significant impact on public health worldwide. This virus is also responsible for occasional outbreaks of pandemic flu, such as the H1N1 in 2009. To date, two classes of drugs are approved for the treatment of Influenza A infection. However, the M2 ion channel blocker (e.g., Rimantadine or Amantadine) is of limited use, because of the rapid emergence of resistant strains and the risk of serious side effects [1], [2]. The resistance to oseltamivir, a widely used neuraminidase inhibitor (NAI), was reported during the 2007–2008 influenza season and since then, a resistant influenza A (H1N1)pdm09 strain harboring an H275Y substitution in NA has emerged [3]–[5]. Recently, increased infections with an oseltamivir-resistant influenza A (H1N1)pdm09 strain have been reported in immune-compromised patients who have not been previously treated with oseltamivir, indicating that this resistant strain has become transmissible [6]–[8]. In addition to this, side effects on the central nervous system have been also reported [9], [10]. Therefore, it is urgent to develop novel strategy to safely and effectively treat influenza virus infection.

Currently, there are several clinical trials on going using the neuraminidase inhibitors (e.g., Peramivir and Laninamivir) [11], [12]. In addition, inhibitors of RNA replication (e.g., Favipiravir) or NS1 viral protein has been under development as effective therapeutic candidates [2], [13]. However, it is quite challenging to develop drugs that are effective and safe on the systemic level, mainly due to the rapid alterations of influenza genome and complicated interactions between virus and host immunity [14], [15].

Influenza is an infectious particle with 8 single-stranded, segmented RNA fragments. The infectivity and transmission of influenza depends on the interaction between its own viral proteins and the host factors that are involved in the anti-viral innate and adaptive immunities. Specifically, the interaction between the viral surface protein HA and the sialic acid receptors in the host surface membrane plays an essential role in the viral entry into the cell through the endocytic vesicle [16]. The virus ribonucleoprotein complexes (vRNPs) are released into the cytoplasm through an M2 ion channel and then transported into the nucleus. The vRNPs are transcribed and replicated by trimeric viral RNA polymerase (PB1, PB2, and PA) and the M1-NS2 complex mediates the viral assembly. The NS1 viral protein inhibits the RIG-I-like receptors (RLRs) innate signaling pathways that recognize the cytosolic viral replication and activate the production of the type I interferons (IFN, including IFNα and IFNβ).

The secreted IFNs act on the neighboring cells and stimulate their signaling pathways for the induction of various IFN-stimulated genes (ISGs), which play key roles in anti-viral innate immunity. The IFN-induced MxA traps viral particles, thereby, repressing the generation of new influenza virus [17], [18]. Meanwhile, type I IFN heterogeneity in the RNA virus infected mammalian cells has been reported and proposed as a balancing mechanism between anti-viral immunity and defense against cell damage [19]–[23]. In the virus-infected host cell population, only a small portion of the infected cells produce IFNs (i.e., become “activated”), which were prone to apoptosis. Whereas, other cells with virus infection remain non-responsive, as they produce none or low levels of IFNs and were unlikely to undergo apoptosis [24].

In development of effective drugs against influenza infection, the construction of a mathematical model that considers the core interaction units between the virus and the infected host cells is helpful in understanding and predicting the systemic outcome of the infected host cell population. For example, the viral production of a pandemic (H1N1 2009) strain in human epithelial cells is intensely and persistently increased compared to a seasonal strain and the elevated replication efficiency was proposed as an underlying mechanism of differential kinetics [25]. The mathematical model focusing on the type I IFN signaling pathway investigated the effect of IFNβ pretreatment on influenza virus-infected host cells and demonstrated that two distinct phases can be identified based on the time delay of IRF7 activation [26]. However, the quantitative analysis of viral kinetics and the dynamics of infected cell population is limiting, although it may be beneficial in verifying key control steps and evaluating the consequences of alterations in each step [27], [28].

Based on the improved understanding of the innate immunity of influenza A virus-infected host cell systems, we attempted to identify the key steps for an effective regulation of influenza production. Consequently, perturbation simulations of the interconnected model of the viral life cycle and the innate defense system were performed. The biological interpretations of the proposed strategies that are suggested by this work present novel insights on advanced treatments against influenza.

## Methods

### Model Equations

**Figure 1:**
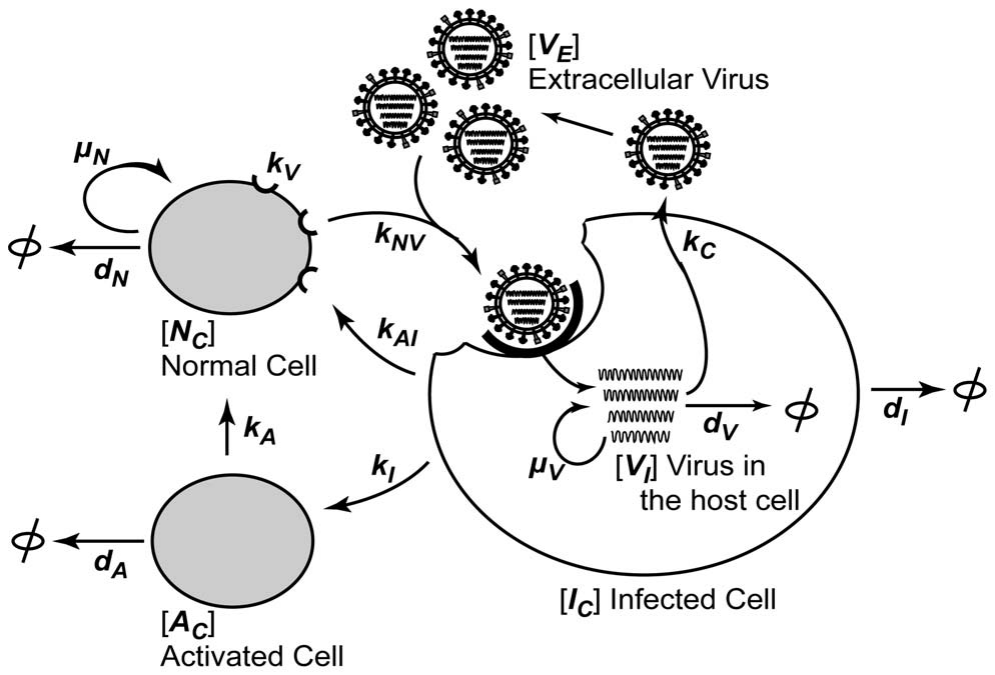
A schematic diagram of the model system. Based on the interaction between the influenza virus and the host cells, three states of host cells (*N_C_*, *I_C_*, and *A_C_*) and two types of viruses (*V_E_* and *V_I_*) were expressed with 13 parameters. For details, see Methods and Table 1.

We constructed deterministic differential equations based on the interconnections between the viral life cycle and the host cell immunity (see Figure 1). First, the influenza virus was classified as either extracellular virus (*V_E_*), which can infect normal cells, or intracellular virus (*V_I_*), which can replicate itself in the host cell. Second, the cells were divided into three types: 1) normal cells (*N_C_*) that can replicate and be infected by virus (*V_E_*), 2) infected cells (*I_C_*), which have been infected by virus, and 3) activated cells (*A_C_*) that express antiviral cytokines, such as IFNβ.
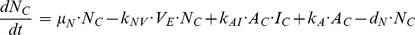
(1)

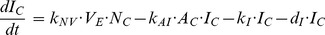
(2)

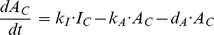
(3)


The amount of normal cells (*N_C_*) increases with the cell growth rate *µ_N_* and decreases with the death rate *d_N_*. The growth rate *µ_N_* was restricted according to the contact inhibitory effect of the cell culture conditions. The normal cell growth was arrested when the total number of cells (*N_C_+I_C_+A_C_*) reaches the maximum confluency (*C_max_*); in all other cases, the cells increased with the maximum growth rate, *µ_N,max_* [29].
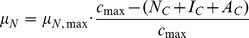
(4)


The normal cells decrease according to the virus infection with a rate of *k_NV_* and increase by the recovery of infected cells with a rate of *k_A_*, which is proportional to the number of activated cells. The infected cells increase with the same rate of *k_NV_*, the infection rate of normal cells, and decrease with the infected cell death rate, *d_I_*. We assumed that the infected cells would become normal cells in proportion to the number of activated cells (*A_C_*), with a recovery rate of *k_AI_*, based on the antiviral functions of IFNβ secreted from the activated cells [30], [31]. Since the heterogeneous IFNβ production in virus-infected cells has been reported in various virus infections [19]–[23], we considered this cellular factor in our model. The infected cells are converted to activated cells depending on the intracellular viral load (*V_I_*/(1+ *I_C_*)), with a specific transition rate *k_I,rel_*.
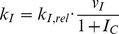
(5)


The activated cells are produced by the transition of infected cells and decrease with their own death rate *d_A_*.
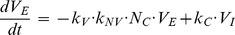
(6)

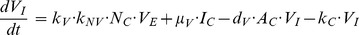
(7)


An extracellular virus particle is attached to the normal cell with the rate *k_V_* and internalized with the rate *k_NV_*. The intracellular virus is increased by this internalization, proliferates with the replication rate *µ_V_*, degraded with the rate *d_V_*, which depends on the cytokine activity that is secreted by the activated cells, and is released with the rate *k_C_*. The amount of extracellular virus increases through the ejection of virus from the infected cell. The nonlinear equations were numerically solved by the standard procedure *ode45* in MatLab® with the initial conditions indicated in the experimental methods.

### Parameter Estimation

Our model incorporates five variables and 13 parameters (see Figure 1 and Table 1). The parameters related to the cellular life cycle, such as the cell replication and the death rate, were set to the values reported in a similar ODE model of influenza-infected human host cells [29], [32]. The values of the parameters that may vary in different influenza strains, were determined through an optimization process using reported experimental data sets for pandemic and seasonal influenza infections [25]. The values of the remaining parameters, such as the recovery rate of infected cells (*k_AI_*) and activated cells (*k_A_*) were set to physiologically reasonable low values. The optimization was performed to minimize the extended least squares of the difference between the influenza dynamics of the experimental data and the simulations using the standard procedure *fmincon* in MatLab®.

**Table 1:** Descriptions and values for the model parameters.

Parameter	Meaning	Value	Unit	Reference
*µ_N,max_*	Maximum growth rate of normal cells	0.03	h^−1^	[29], [32]
*k_NV_*	Virus internalization rate	0.0014	pfu^−1^·h^−1^	[29], [32]
*k_AI_*	Recovery rate of infected cells	0.000001	cells^−1^·h^−1^	
*k_I,rel_*	Transition rate of infected cells to immune-activated cells	0.0102	h^−1^	[25]
*k_A_*	Recovery rate of activated cells	0.000001	h^−1^	
*µ_V_*	Viral replication rate : seasonal	0.00041	pfu·cells^−1^·h^−1^	[25]
*µ_V_*	Viral replication rate : pandemic	0.0131	pfu·cells^−1^·h^−1^	[25]
*d_V_*	Viral degradation rate	0.00001	cells^−1^·h^−1^	[25]
*d_N_*	Death rate of normal cells	0.001	h^−1^	[29], [32]
*d_I_*	Death rate of infected cell	0.001	h^−1^	
*d_A_*	Death rate of activated cells	0.0257	h^−1^	[29], [32]
*k_C_*	Viral release rate	9.996	h^−1^	[25]
*k_V_*	Viral attachment rate	1	pfu·cells^−1^	
*C_max_*	Maximum cell confluency	10000000	cells	

## Results

### Host Cells Infected with Pandemic Influenza Exhibited more Severe Progress of Infection than Cells Infected with Seasonal Influenza

**Figure 2:**
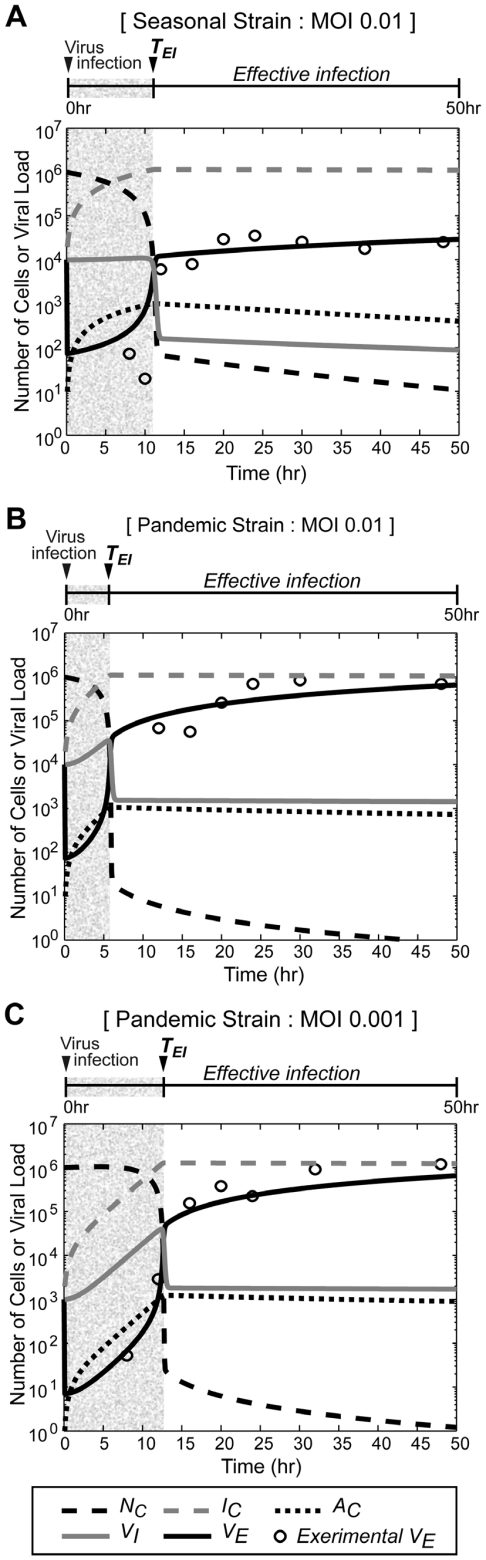
Quantitative dynamics of host cells infected with the seasonal and pandemic strains of influenza virus. The experimental values of viral loads (*circle*) produced from the infection of human bronchial epithelial cells were used for the parameter estimation [25]. The simulated dynamics of the extracellular virus (*solid black line*), intracellular virus (*solid gray line*), normal cells (*long dashed black line*), infected cells (*dashed gray line*), and activated cells (*short dashed line*) are presented for different influenza strains and initial conditions: (**A**) Influenza A/NewCaledonia/20/99 (10^4^ pfu) with an initial 10^6^ normal cells; (**B**) Influenza A/California/04/2009 (10^4^ pfu) with 10^6^ normal cells; (**C**) Influenza A/California/04/2009 (10^3^ pfu) with 10^6^ normal cells. For simulation of viral dynamics in A, B, and C, 9, 6, and 7 experimental data points were used respectively in the parameter estimation. The *T_EI_* indicates the time required to reach effective infection. The detailed parameter values used in this simulation are shown in Table 1.

We constructed a mathematical model focused on the interaction between the influenza virus and the host cells whose multiple interactions were expressed as a set of differential equations (Figure 1. For detailed description, see Methods). To determine the model parameters of the pandemic and seasonal influenza viruses, we used previously reported influenza dynamics of a seasonal (A/NewCaledonia/20/99) and a pandemic strain (A/California/04/2009), which were obtained in differentiated human bronchial epithelial cells (Table 1). The only difference in the parameter values that differentiate the seasonal and pandemic influenza strains was the viral replication rate (*µ_V_*), which indicates that the intrinsic nature of the intracellular viral replication significantly affects the pathogenicity of pandemic strains. The goodness-of-fit for the optimized results was verified with a κ^2^ test for each experimental data: the seasonal strain with an MOI of 0.01 (Figure 2A: p<0.02), the pandemic strain with an MOI of 0.01 (Figure 2B: p<0.07), and the pandemic strain with an MOI of 0.001 (Figure 2C: p<0.03). The two simulated dynamics of pandemic (Figure 2B) and seasonal (Figure 2A) strains were significantly different from each other (ANOVA test : p<0.0001).

At 50 hours after the infection, levels of both the extracellular and the intracellular virus particles were found to be approximately twenty times higher in the cells infected with pandemic strain (Figure 2A–D). The simulated dynamics of normal cells (*N_C_*)_,_ activated cells (*A_C_*)_,_ and infected cells (*I_C_*) showed that the levels of normal cells were significantly different between these two stains. In both strains, a rapid reduction in the number of normal cells was observed when the number of extracellular virus dramatically increased. Since this pattern was observed only when the infected virus was successfully replicated within the host cells, the amount of time elapsed until the fall of N_C_ and the increment of *V_E_* crossed each other reflects the time that is required to get effective infection, *T_EI_*.

With the same MOI, the pandemic virus exhibited a *T_EI_* that was half that of the seasonal strain. Although the number of *I_C_* and *A_C_* remained relatively unchanged, the number of *N_C_* with the pandemic virus infection was approximately 10 fold less than the number of those that had obtained with the seasonal virus infection during the period of effective infection. In fact, the number of *N_C_* in the pandemic infection actually reaches zero, which indicates that every cell is either infected or dead. With a ten-fold lower dose of the pandemic virus (MOI = 0.001), the *T_EI_* was increased more than two fold. However, after 50 hours of infection, the *I_C,_ N_C_* and *A_C_* were not significantly changed when the extracellular virus reached its saturation level in both in the simulation and the experiments (Figure 2D).

**Figure 3:**
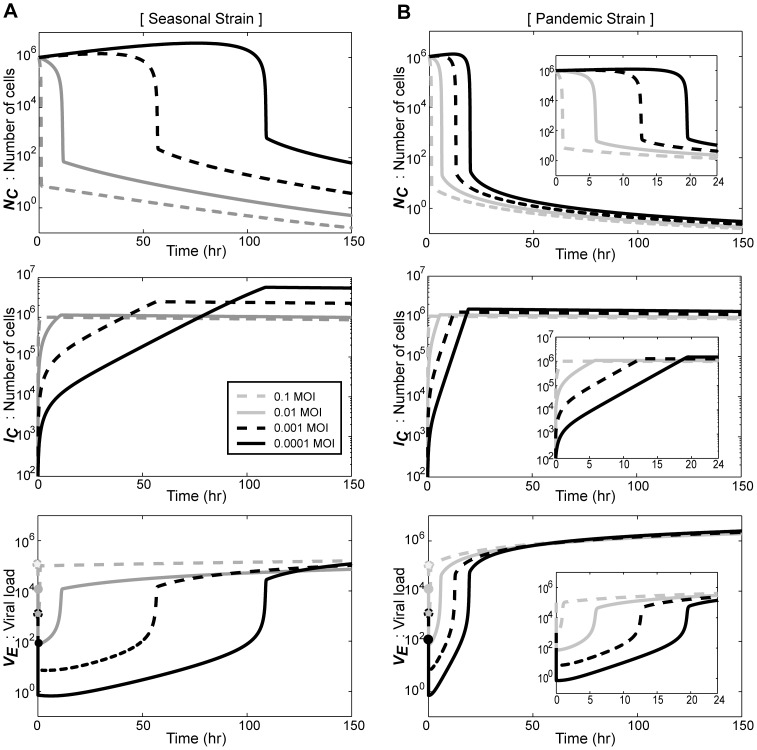
Dynamics of cells and virus with varying initial doses of the seasonal and pandemic strains. The dynamics of *N_C_*, *I_C_*, and *V_E_* were simulated for 150 hours after infection, with varying MOIs (0.0001–0.1) for infection with seasonal (A) and pandemic (B) influenza strains. The dynamics of each variable for the first 24 hours of infection are displayed within the small boxes.

Therefore, we systemically examined the effect of the initial number of pandemic and seasonal virus particles on the dynamics of the three different host cell states during the course of infection. The model system was numerically simulated with varying viral load (*V_E_*) at t = 0 in the range of 10^2^–10^5^ (which corresponds to MOIs in the range of 0.0001–0.1). In both strains, the time to reach effective infection was prolonged, with smaller dose of the initial infection. The accumulation of the extracellular virus (*V_E_*), as well as the number of infected cells (*I_C_*), was severely delayed in the course of the seasonal influenza infection, which indicates that either the intrinsic rate of the viral replication or the viral release, in addition to the differential induction of the innate anti-viral immunity of the host cells might be responsible for these slack responses. As a result, the *N_C_* in the population with seasonal virus infection remained high for a relatively long time; in addition, even after the onset of effective infection, substantial amounts of *N_C_* were still observed (Figure 3A). In contrast, the population with pandemic virus infection attained effective infection much faster than that the population infected with the seasonal strain (Figure 3B). Furthermore, the *N_C_* in the population infected with the pandemic virus strain decreased rapidly and reached an insignificant number during the period of effective infection. In summary, the host cells infected with the pandemic strain exhibited a more aggressive progression for broader range of initial viral loads compared with the seasonal influenza strain.

### Viral Release Rate (*k_C_*) is the most Influential Factor for Influenza Production

**Figure 4:**
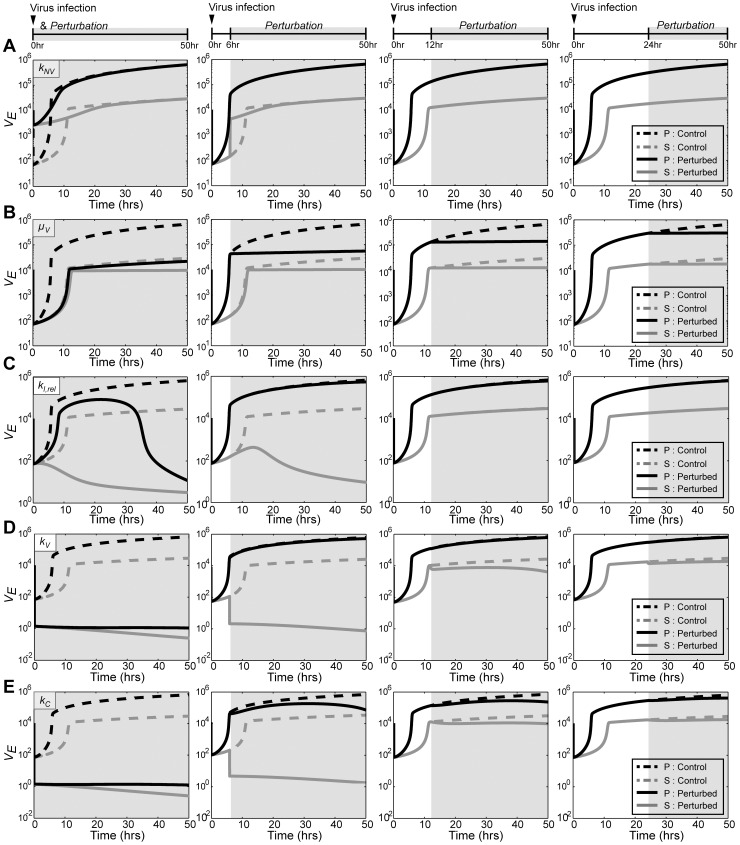
Effect of parameter perturbations on the production of influenza virus. Each parameter was numerically perturbed 0, 6, 12, or 24 hours after infection, and the dynamics of *V_E_* were simulated for a total 50 hours. The internalization rate *k_NV_* (A), the replication rate *µ_V_* (B), and the release rate *k_C_* (E) were reduced 50-fold, whereas the transition rate *k_I,rel_* (C) and the attachment rate *k_V_* (D) were increased 50-fold. The dynamics of the seasonal strain (*gray solid line*) and the pandemic strain (*black solid line*) without any perturbations were compared with those of the perturbed simulations of the seasonal (*gray dotted line*) and pandemic (*black dotted line*) strains.

Based on the model simulations of the virus-host interactions in the seasonal and pandemic influenza infections, we next attempted to identify the key controlling parameters that minimize the viral production and promote the survival of normal cells. For this purpose, changes in the dynamics of *V_E_* were examined with single parameter perturbations during the seasonal and pandemic influenza infections (Figure 4). Each parameter was numerically altered (either reduced 50-fold (*k_NV_*, *µ_V_*, and *k_C_* ) or induced 50-fold (*k_I,rel_* and *k_V_*) relative to their original values) 0, 6, 12, or 24 hours after infection, and the dynamics of *V_E_* were analyzed for a total of 50 hours after infection. Among the parameters tested, the internalization rate of virus (*k_NV_*) exhibited the least effect on the dynamics of *V_E_* (Figure 4A). The perturbation of the viral replication rate (*µ_V_*) effectively inhibited the pandemic, but not the seasonal influenza strain only when it was perturbed during the early stages of the infection (Figure 4B). The most effective parameter perturbations (*k_I,rel_*, *µ_V_*, *k_V_*, and *k_C_*) exhibited weakened inhibitory effects when the perturbation were performed at later stages of the infection.

The most dramatic effect on the dynamics of *V_E_* was observed with perturbations on the intracellular immunity (*k_I,rel_*), the attachment ratio of the virus to the cell (*k_V_*), and the release rate of the virus (*k_C_*), although their effect was also sensitive to the time of the perturbation (Figure 4C–E). The overall simulation for a single degree of parameter perturbations showed that *k_I,rel_*, *k_V_*, and *k_C_* are critical parameters for the effective manipulation of the viral production.

**Figure 5:**
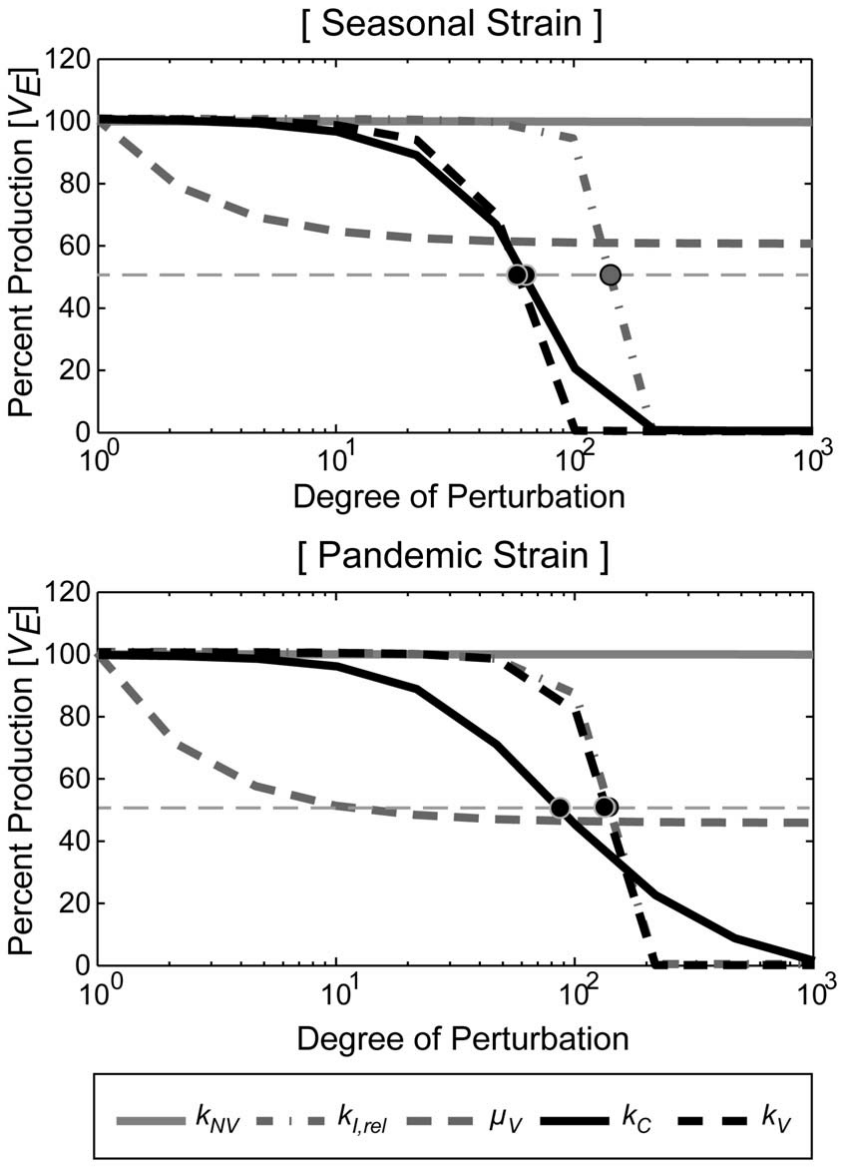
Kinetic analysis of parameter perturbations on the influenza production. For the seasonal and pandemic strains, the kinetic behaviors of each parameter perturbations were simulated. The parameter perturbations were performed 24 hours after infection, and the relative reduction in the viral production compared to the control simulations were measured after 50 hours of infection. The *thin gray dotted line* indicates the 50% inhibition of the viral production.

To determine the kinetic behavior of each inhibition in the control of the viral production, the selected parameters were further simulated using various degrees of perturbations 24 hours after infection. The resulting *V_E_* values after 50 hours of infection were expressed as percentile scores relative to the value of *V_E_* in the absence of any perturbations (Figure 5). We found that the blockage of the viral replication (*µ_V_*) exhibited the most sensitive effect in the inhibition of the viral production because this production started to decline with a very low degree of inhibition. However, the inhibition effect was quickly saturated at the 60% and 45% level in the seasonal and pandemic strains, respectively. In contrast, *k_C_*, *k_V_*, and *k_I,rel_* block viral production completely, at a higher degree of perturbation. In the simulation of the seasonal strain, the perturbations of *k_C_* and *k_V_* exhibited similar degrees of half-maximal reduction (D_50_) of the viral production (65.2 and 61.5, respectively), whereas the D_50_ of *k_I,rel_* was 153.9. These data indicate that the rate of viral attachment to the host cells and the rate of viral release from the host cells can be effective targets for the manipulation of the production of seasonal influenza virus. In contrast, the viral production of the pandemic strain was similarly affected by perturbations of *k_V_* and *k_I,rel_*, with D_50_ values of 144.3 and 148.7, respectively, whereas the D_50_ of *k_C_* was 90.4. This result indicates that the release process of the newly synthesized pandemic influenza virus particles might be an effective target for manipulating viral production.

### Combinatorial Perturbations of *k_C_* and either *k_I,rel_* or *k_V_* Effectively Suppress Viral Production in Progressed Influenza Infection

**Figure 6:**
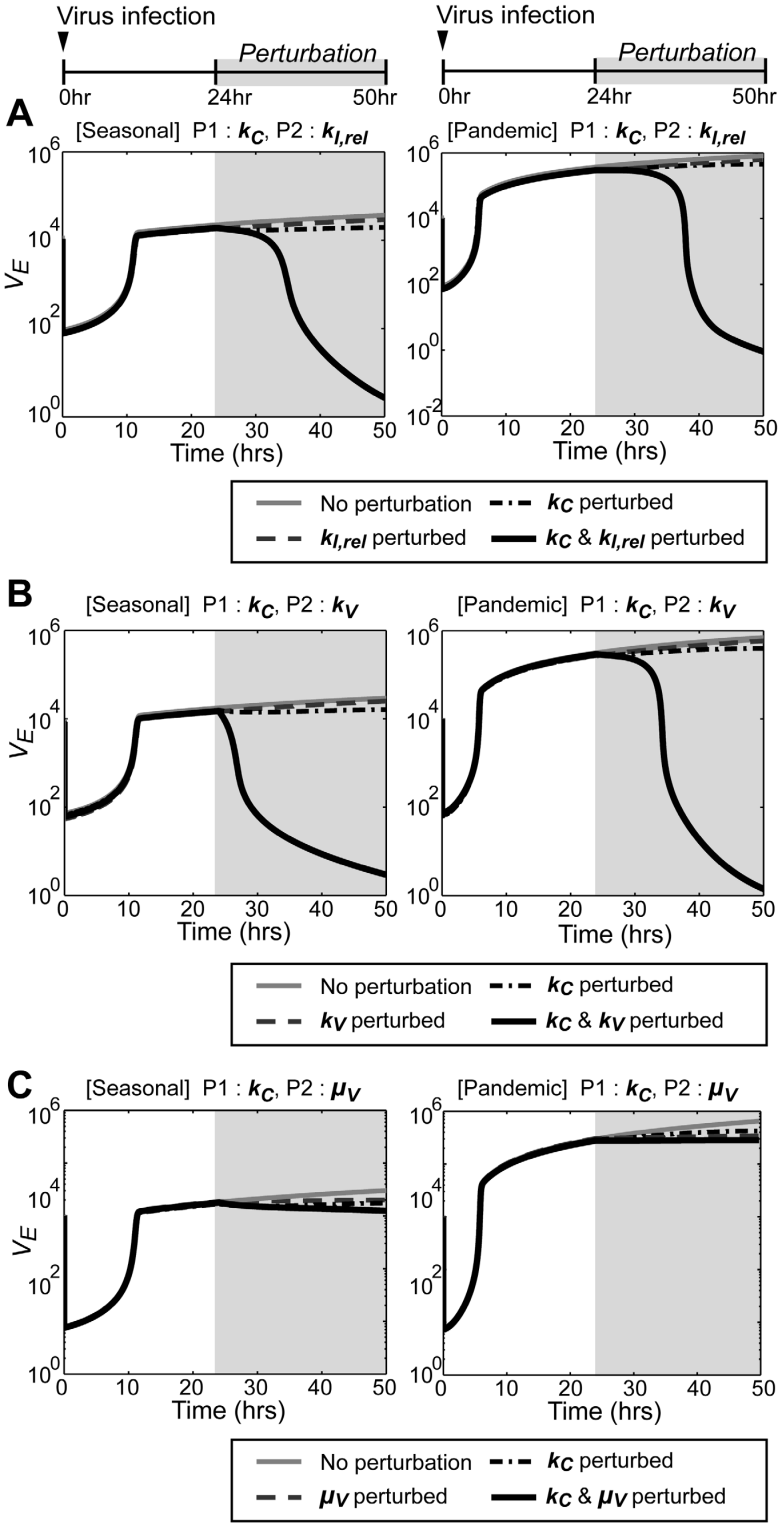
Inhibitory effect of combinatorial perturbations on the viral production of the seasonal and pandemic strains. (**A–C**) The perturbations were numerically performed 24 hours after infection. In addition to the 50-fold reduction of the *k_C_* value, a ten-fold increase of *k_I,rel_* (A) or *k_V_* (B) or a ten-fold reduction of *µ_V_* (C) were applied. The dynamics of *V_E_* as a result of the combinatorial perturbations (*black line*) was compared with those obtained in the absence of any perturbations (*gray line*), or with a single perturbation of *k_C_* (*black dotted line*), *k_I,rel_*, *k_V_*, or *µ_V_* (*dark gray dotted lines*).

To clinically treat influenza infection, it is critical to achieve complete suppression of influenza production after the progression of effective infection in the host cells. Although several drugs are in use or under development for the treatment of influenza infection, the efficacy of these drugs significantly depends on the stage of the viral infection; in addition, influenza often acquires resistance to one or more of these drugs [33]–[35]. Therefore, it is essential to develop a combinatorial treatment strategy that efficiently suppresses viral production even at a later stage of infection. Thus, we examined the effect of perturbing both *k_C_*_,_ which exhibited the most promising effect in our single parameter perturbation simulations, and additional parameters 24 hours after infection (Figure 6). Among the parameters examined, the combinatorial perturbations of *k_C_* and either *k_I,rel_* or *k_V_* proved to be the most effective to suppression of both pandemic and seasonal influenza productions, whereas the other parameters, including *µ_V_*, were hardly effective (Figure 6A–C). Therefore, these data suggest that, when effective viral production is in progress, a superior reduction of the influenza production might be achieved by the blockage of the viral release in addition to the enhancement of either intracellular immune responses or the viral adhesion to host cells.

## Discussion

The infection of influenza A virus is initiated by the attachment of the virion to the surface of the host epithelial cells. The propagation of the infected virus is controlled not only by the intrinsic properties of the viral or host genome but also by the complicated interactions between them [36], [37]. Therefore, it is not easy to predict the outcome of influenza infection and the fates of the infected host cells. To understand the critical steps and the interactions that affect the viral and infected host cell dynamics, it is essential to quantitatively analyze the influenza dynamics and the infected cell population. In this study, through the simulation of simple models of the virus-infected host cell interactions, we attempted to illustrate the characteristics of the cellular and viral dynamics of host cells infected with pandemic and seasonal strains of influenza.

Specifically, we focused on the parameters that significantly affect the time required to achieve effective infection (*T_EI_*) and the dynamics of the normal cells (*N_C_*) during the period of effective infection. After *T_EI_* is reached, a dramatic increase in the extracellular virus (*V_E_*) and a sudden decrease in the amount of normal cells is observed. The *T_EI_* was sensitive to the initial viral load of infection because it took a longer time to reach effective infection with a lower MOI, in both the pandemic and seasonal strains (Figure 3). A similar effect of the MOI on the dynamics of influenza A production has been previously reported in influenza-infected MDCK cells [38].

In our model simulation, we identified several biological parameters that affect the dynamics of viral production; although the only difference between the pandemic and seasonal strains were derived from the viral replication rate (*µ_V_*), the strongest blockage of viral production was achieved by a combinatorial perturbation of the viral release rate (*k_C_*) and either the viral attachment to the host cells (*k_V_*) or the activation of intracellular immunity (*k_I_*). In most of the simulations with single parameter perturbations, the effects on the viral dynamics were significant when the perturbations were performed during the early stages of the infection. This finding is in agreement with the previous study that the addition of T-705, which is a chemical that inhibits viral replication, to MDCK cells infected with the influenza A/PR/8/34 strain is effective only when it is administered within 4 hours after infection [39]. Furthermore, it provides partial explanation for the decreased Oseltamivir efficacy administered later than the first 12 hours of fever onset [36], which can be mimicked when we chose a larger degree of perturbation.

The currently available drugs that are frequently used to treat influenza infection are Oseltamivir, Zaninamivir, Amantadine, and Ribavirin, which have been reported to inhibit viral release, viral internalization, and viral replication, respectively [11], [40]. In the model simulations that compare the degree-dependent kinetics of the single parameter perturbations (Figure 5), the inhibitory effect of the viral release rate (*k_C_*) was the most prominent compared with the viral internalization rate (*k_NV_*) and the viral replication rate (*µ_V_*). In support of our simulation results, Oseltamivir exhibited the lowest halfmaximal effective concentration, EC_50_, compared to the other drugs (Amantadine, and Ribavirin) in MDCK cells infected with the A/NewCaledonia/20/99 influenza virus [41]. Although it is hard to directly compare the efficacy of the different drugs, the data indicates that model simulations might be useful for the investigation of the key biological parameters that affect the viral and host dynamics in populations infected with influenza A virus. Combinatorial treatments of various drugs have been widely studied to efficiently control an infection with influenza virus [42]–[45]. As we predicted, a combinatorial treatment of Oseltamivir with Ribavirin, which inhibits the viral replication, exhibited increased efficacy in MDCK cells infected with A/NewCaledonia/20/99 influenza virus [41].

Perturbations in the cellular processes that control the viral release are an effective way to control the influenza production during the early stages of infection; however, our simulation also proposed two alternate strategies to achieve stronger inhibitory effects at later stages of infection (Figure 6). First, when performed in addition to the reduction of *k_C_*, the reinforcement of the intracellular immunity (*k_I,rel_*) dramatically suppressed the viral production 50 hours after viral infection either strain. Because the innate immunity against influenza virus is mainly mediated by the RLR- IFNα/β signaling pathways, various approaches that activate RIG-I or increase the production of type I IFNs might be used in combination with agents that inhibit the viral release. It has been previously reported that polyI:C, which is a synthetic double stranded RNA that activates RLRs, or RIG-I aptamer, stimulates the intracellular immune responses of infected host cells and efficiently blocks viral production in influenza A/PR/8-infected A549 cells [46]. In a mouse model, the intranasal administration of poly-ICLC also suppressed the virus titers of influenza A/PR/8 virus [47]. Second, an increase in the viral attachment to the host cell surface (*k_V_*) greatly elevated the inhibitory effect of the viral release (*k_C_*) on the dynamics of viral production. It is somewhat puzzling phenomenon and there is little physiological data that support this speculation. However, it is noteworthy to mention that certain mutant strains of the Japanese encephalitis virus, the Murray Valley encephalitis virus, and the Venezuelan equine encephalitis virus that show a stronger binding ability to the host cell surface membrane exhibit attenuated virulence or accelerated clearance compared to the parental strains [48], [49]. Therefore, it might be interesting to further investigate whether there is a negative correlation between the binding affinity to the host cell surface and viral production.

In summary, we developed a model system to analyze the quantitative dynamics of an influenza A-infected host cell system and proposed a novel strategy to effectively control the viral production in the infected host. Despites its value, our model simulation has several limitations. First, in this model analysis, we mainly focused on the immediate early kinetics of virus and infected cells, *in vitro*. During the *in vivo* infection of influenza virus, however, the primary infection occurs in the surface of respiratory tract, and diverse innate and adaptive immune cells are recruited to control the infection [28], [50]. Second, in our simulation, we used a fixed degree of perturbation to measure the effect. However, local concentrations and the efficacy of the treated drugs are expected to vary *in vivo*, in a spatial and temporal context dependent manner. Therefore, this model may only be useful for interpreting the early dynamics of influenza and cellular response occurring in the limited space of infection, whose quantitative dynamics is barely measured from *in vivo* experiments. Nonetheless, it would be of great interest to apply these findings to identify the main biological factors that control the binding affinity of influenza to the host cell in the future.
